# Seasonal variation in the metabolome expression of *Jania rubens* (Rhodophyta) reveals eicosapentaenoic acid as a potential anticancer metabolite

**DOI:** 10.1038/s41598-023-42497-0

**Published:** 2023-09-20

**Authors:** Nimrod Krupnik, Alvaro Israel, David Meiri

**Affiliations:** 1 Faculty of Biology, Technion, 32000 Haifa, Israel; 2 Israel Oceanographic & Limnological Research, The National Institute of Oceanography, Tel Shikmona 8030, 31080 Haifa, Israel

**Keywords:** Cancer, Drug discovery, Plant sciences, Natural products

## Abstract

Seaweeds of the intertidal zone are subjected to diverse stresses due to environmental changes in radiation, salinity, water quality, herbivore communities, etc. Thus, marine seaweeds developed various unique compounds to deal with environmental fluctuations. Therefore, they are a good source of unique novel compounds. Here, we explored the seasonal metabolomic changes in *Jania rubens* and found notable changes between extracts of different seasons in the metabolomic profile and in their anticancer activity. The most bioactive extract was from samples collected during the Fall season, which demonstrated an LC50 of 178.39 (± 10.02 SD) µg/ml toward Non Small Cell Lung Cancer (NSCLC) followed by the Winter season extract. The Fall and Winter extracts also displayed more resemblance in their metabolic profile relative to Spring and Summer extracts. The Fall extract was fractionated and tested for cytotoxic activity toward an array of cancer cell lines. Eventually, using a bio-guided assay and multiple fractionation steps, we isolated and identified the essential fatty acid, eicosapentaenoic acid, as the active anticancer agent, showing an LC50 of 5.23 (± 0.07 SD) µg/ml toward NSCLC. Our results emphasize the potential use of *J. rubens* as a source of beneficial fatty acids and stress the importance of environmental effects on metabolic constitutes.

## Introduction

Seaweed extracts and their isolated natural products are gaining popularity in many aspects of healthcare including cosmetics and pharmacology. Their potential uses are investigated for a variety of applications such as anti-inflammatory^1^, anti-bacterial, wound healing^2^ and anti-cancer^3^ effects. Seaweeds contain a broad range of compounds with pharmaceutical use from diverse chemical classes such as polyphenols, polysaccharides, pigments, and Poly Unsaturated Fatty Acids (PUFAs)^4,5^. For example, pheophorbide, a product of chlorophyll degradation that was isolated from the Red Seaweed *Grateloupia elliptica,* has shown anti-cancer activity in glioblastoma cell lines^6^. Fucoxanthin, extracted from *Laminaria Japonica* has demonstrated reduced metastasis and even increased sensitivity to chemotherapy drugs by lung cancer cells^7^. Overall, there is numerous evidence suggesting marine seaweed-originated compounds with biological activity for therapeutic purposes^4,5,8–10^.

Naturally occurring seaweeds are subjected to major environmental shifts as a result of seasonal changes in light radiation, temperature, salinity and other factors. Moreover, herbivore communities’ changes and even increase in runoff pollution near shores during the rainy seasons lead to chemical changes^11,12^. To thrive under changing conditions, seaweeds, namely of the intertidal zone, respond by means of chemical defense using a vast array of metabolic compounds^13,14^. It was previously shown these seasonal shifts affect the algal metabolic profile^13,15^ and, as a result, their biological activity^16,17^.

*J. rubens* (*Rhodophyta*), a calcified alga of the order *Corallinales*, is considered one of the most common seaweeds in the Eastern Mediterranean intertidal zone^18^. *J. rubens* is characterized as a small branchy seaweed with thin, segmented, dichotomous, cylindrical thalli, forming dense mats covering the abrasion platforms with colors ranging from dark red to complete white under bleaching conditions during low tides. It can be found attached to the substrate or as an epiphyte that grows on other seaweeds.

*J. rubens* is currently under investigation for its anticancer activity. Anti-tumorigenic effects of *J.rubens* extracts have been previously reported by several different studies^19–22^. A polysaccharide extract from the seaweed was shown to negatively affect breast and colorectal cancer cell proliferation^22^. Methanolic extracts have displayed growth inhibition properties on hepatoblastoma cells in-vitro and Ehrlich ascites carcinoma (EAC) cells in in-vivo^21,23^. Ktari et al.^19^ have studied the cytotoxic effect of a *J. rubens* extract on mouth epidermal carcinoma cells, which resulted in the isolation of an active sterol. A few years later, seven types of brominated diterpenes were isolated from the same alga after presenting some anticancer properties on EAC cells^24^.

Taking together the anticancer potential of *J. rubens* secondary metabolites along with the environmental annual changes in its costal habitat, we hypothesized that the seasonal environmental changes in the Eastern Mediterranean coastline throughout the year may dramatically affect the metabolomic constitutes and cytotoxicity of the seaweed. We set out to investigate which metabolites that demonstrate seasonal variation lend the seaweed its cytotoxic activity.

## Materials and methods

### Reagents

Mass spectrometry (MS)-grade acetonitrile, methanol (MeOH), and water were used for the mobile phases, and high-performance liquid chromatography (HPLC)-grade MeOH, water and ethanol were used for sample preparation. All were obtained from Mercury Scientific and Industrial Products Ltd. (Rosh Haayin, Israel). LC/MS grade acetic acid (AcOH) and serum (S1-100 mL) for method validation were purchased from Sigma-Aldrich (Rehovot, Israel). Ecosepantonoic acid (EPA) and PUFA mixed standards were purchased via Cayman chemicals (Michigan, United States), products numbers 90110 and 17941, respectively.

### Seaweed sampling

*J. rubens* (IOLR herbarium access number 00401) samples were collected along the Israeli Mediterranean coast line: Achziv beach (33.043111° N, 35.099028° E), Shikmona marine reserve in Haifa Bay (32.826056° N, 34.956° E), Habonim beach (32.630194° N, 34.920444° E) and Herzliya beach (32.139612° N, 34.789362° E), starting January 2020 through May, August, and November of the same year. Samples were collected in bags containing seawater and kept on ice in a cooler until reaching the lab for further process. Samples were identified according to the local seaweed lexicon by Dr. Rachel Einav^18^ with the help of Dr. Israel Alvaro and Dr. Tamar Guy-Haim from the Israeli Oceanography and Limnological Research (IOLR) center.

### Sample preparation and extraction

Samples were washed with fresh water, manually cleaned from epiphytes and sand, frozen at -80°C followed by lyophilization (Labogene, Denmark) to complete dryness and stored at -80°C. The dried samples were ground to a fine powder using a generic spice grinder (Morphy Richards, UK), then further sifted with a metal 1 mm mesh size strainer to remove inorganic particles. Exactly 50 g of the powder were accurately weighed and extracted with 5 L of (1:100 w/v) under stirring for 5 h. Samples were then filtered once through Whatman filter paper number 4 and once through Whatman 0.22 µm nylon membrane. The crude extract was evaporated under vacuum (Heidolph, Germany) at 39 °C to complete dryness, weighed, and resuspended with either MS-grade MeOH, chloroform:MeOH (2:1 v/v) for the fractionation process or MS-grade dimethyl sulfoxide (DMSO) at a concentration of 50 mg/ml for biological assays.

### Chromatographic separation

100 g of reversed phased (RP) silica (LiChroprep® RP-18 (40–63 µm), Merck, Germany, Cas NO. 108688-10-4) was placed in a glass column coupled to a vacuum pump (Vacuubrand, Germany) for Vertical Liquid Chromatography (VLC). After excitation and conditioning using 300 ml MeOH followed by 300 ml H_2_0, 2 g of the crude *J. rubens* chloroform:MeOH (2:1 v/v) extract were applied and elution was carried with 1 L of solvent with increased ratio of MeOH up to 100%, followed by chloroform:MeOH (2:1, v/v), and ethyl acetate. The resulting fractions were evaporated under vacuum at 39 °C to complete dryness, weighed and resuspended with MS-grade MeOH. The active 80% MeOH (VLC80) product underwent further fractionations using Thermo Scientific semi-preparative UHPLC system, coupled with an automated fraction collector and a UV detector set to 223 nm, through Luna® C18(2) (10 μm, 250 mm × 21.2 mm) column with a guard column (15 μm depth filter × 21.2 mm) (Phenomenex, Torrance, CA, USA). Three fractionation steps were applied with H_2_O for solvent A and MeOH for solvent B, both added with 0.1% acetic acid modifier. The VLC80 was fractionated into four fractions by the following method: min 0–5, 60% B; min 32–37, 97% B; min 37.1–39 60% B. Fractions were collected at 9 min intervals after 3 min from run start. Second step fractionation was carried for the active fraction 4 (VLC80F4) to create 3 subfractions: min 0–5, 90% B; min 30–35, 97% B, min 35.1–36, 90% B. Fractions were collected at 11 min intervals after 3 min from run start. Third step separation of the active fraction 1 (VLC80F4-1) was conducted to obtain 3 subfractions as follows: min 0–5 90% B; min 30–35 92% B; min 35.1–36 90%B. Fractions were collected for 11 min intervals after 3 min from run start. All separation methods were conducted at a flow rate of 15 ml/min.

### Cell lines

All cell lines were purchased from the American Type Culture Collection (ATCC); A549 (CAT NO. CCL-185), SW480 (CCL-228), lnCAP (CRL-1740), and PC3 (CRL-1435) cell lines were maintained using RPMI-1640 medium (Sigma-Aldrich, R8758). NCI-H460 (HTB-177), HT29 (HTB-38), MCF7 (HTB-22), and MDA-MB-231 (HTB-130) cell lines were kept in high glucose DMEM (Sigma-Aldrich, D5796). Media were supplied with 10% FBS (Biological Industries, 04-007-1A), 100 units/ml of penicillin G and 100 μg/ml of streptomycin (Biological Industries, 03-031-1B). Normal bronchial/tracheal epithelial Cells (PCS-300-010) were grown in serum-free Airway Epithelial Cell Basal Medium (ATCC, PCS 300-030) supplemented with the appropriate cell growth kit (ATCC, PCS-300-040). All cells were maintained in a humidified atmosphere of 5% CO_2_ at 37 °C.

### Viability assay

Cell survival assays were conducted as described in Baram et al.^25^. Briefly, 1 × 10^4^ or 8000 (for A549) cells/well were cultured in 96-well plates and incubated overnight followed by the replacement of the growth medium with fresh media containing 0.5% FBS. Seaweed extracts in DMSO, at a concentration of 50 mg/ml, were diluted to 1 mg/ml in PBS (Sigma-Aldrich, D8537) and added to the cells as indicated. Cells were incubated with treatments for 48 h, then stained using the fluorescent probes propidium iodide (PI) (Sigma-Aldrich, P4864) and Hoechst (Thermo Fisher Scientific, H3570). Cells were visualized using an ImageXpress Micro® system (Molecular Devices, Sunnyvale, CA, USA). Four sites were imaged in each well and the number of detected signals per well was counted and analyzed by MetaXpress® software (Molecular Devices). The following formula was applied to calculate the rate of cell death:

$$\mathrm{\% \,Cellular \,\,death }= \frac{(Tc-Ts)+Ds}{TC}\times 100$$

where Tc- is the total number of cells in equal concentration in the control (DMSO), Ts is the total number of cells in the sample treatment, and Ds is the total number of dead cells in the sample treatment.

### Flow cytometry cell viability

Annexin V/PI staining was used to determine the apoptotic rate. A549 cells were cultured in 6-well plates at a concentration of 1 × 10^5^ cells/well. After 24 h of incubation the media was replaced with fresh media containing 0.5% FBS, and each well was treated with appropriate extract and further incubated for 24 h. Treated cells were washed, collected and resuspended with annexin binding buffer (BioVision, Milpitas, USA) containing Annexin V-FITC (BioLegend, San Diego, USA) and PI (BioLegend, San Diego, USA) (1:1 v/v). Measurements of Annexin (R 660/20nm) and PI (B 575/26nm) were obtained using BD™ LSR II digital four-laser flow cytometer (BD Biosciences) and were analyzed by BD FACSDiva™ software version 6.1.2. (BD Biosciences).

### Metabolites identification with UHPLC-HRMS

Samples were filtered through a 0.22 µm PTFE syringe filter and diluted to 10 mg/ml in MS-grade MeOH prior to analysis. The chromatographic separation was performed in Data-dependent MS/MS mode as described by Berman et al.^26^ with several modifications. The process was carried out using a Thermo Scientific UHPLC system coupled with a Q Exactive™ Hybrid Quadrupole-Orbitrap MS (Thermo Scientific, Germany). The chromatographic separation was achieved using Luna® C18(2) (3 μm, 150 mm × 4.6 mm) with a guard column (0.5 μm depth filter × 0.1 mm) (Phenomenex, USA) and a multistep gradient (solvent A: Milli Q water containing 0.1% acetic acid, solvent B: methanol containing 0.1% acetic acid. All solvents were of MS-grade).

The multistep gradient program was established as follows: initial conditions were 75% B for 3 min, raised to 90% B until 3.1 min and maintained for 10 min. Then elevated to 95% B until 10.1 min and held at 95% B until minute 15, and decreased to the initial condition of 75% B until minute 20, for re-equilibration of the system prior to the next injection. A flow rate of 0.25 ml/min was used, the column temperature was 30 °C and the injection volume was 1 μL. MS acquisition was carried out with a heated electrospray ionization (HESI-II) ion source operated in negative mode. Source parameters were as follows: sheath gas flow rate, auxiliary gas flow rate and sweep gas flow rate: 10, 20 and 1 arbitrary units, respectively; capillary temperature: 350 °C; heater temperature: 50 °C; spray voltage: 3.6 kV. The scan range was 120–1000 m/z for all acquisition events. Data acquisition in full MS1 mode was performed at 70,000 resolutions, and the AGC target was set to 1e6 with a maximum IT of 100 ms. Compound identification was conducted by comparison to the National Institute of Standards and Technology (NIST) library (version 2.2).

### Chemometrics

MS/MS feature detection and quantification were performed using MZmine2 software^27,28^. The MZmine2 workflow was executed with Ubuntu 2.04.4 LTS 64bit OS running on a computer with dual E5-2640 Intel Xeon. Parameters for MZmine processing were set as follows: noise level 2E4, chromatogram builder, using Automated Data Analysis Pipeline (ADAP), conducted using minimum group size of 5 above 2E4 threshold with a minimum highest intensity of 4E4 and an m/z tolerance of 5 ppm. Deconvolution of the chromatogram was conducted using the ADAP presetting, m/z range of 0.01 and retention time (r.t.) range of 0.1. Group isotope, using the isotopic peak grouper, was conducted with m/z tolerance of 5 ppm, r.t. tolerance of 0.1 and a maximum of 3 charges. Feature alignment using r.t. tolerance of 0.1, m/z weight of 0.75 and r.t. weight of 0.25. The feature list was filtered using join list row filter, keeping peaks with MS2 data only and gap filling with intensity tolerance of 10%, m/z tolerance of 5 ppm and r.t. tolerance of 0.2. For NMDS analysis, features found in blanks as higher than 2% of the mean amount of the feature quantity in all samples were omitted.

### Statistics

All statistical analyses and models were executed using JMP15 (SAS Institute, USA), and R software (R Development Core Team, 2021) using, “ecodist”^29^ and “vegan”^30^ packages. LC50 was calculated using 3 parameters logistic on log y scale. Data are reported as the mean ± SE of at least three independent experiments. Multiple groups were compared using one-way or two-way ANOVA followed by a Tukey HSD posthoc multiple comparisons test. P values were adjusted using the Bonferroni correction method and a value of P ≤ 0.05 was considered significant for all tests.

## Results

**Figure 1:**
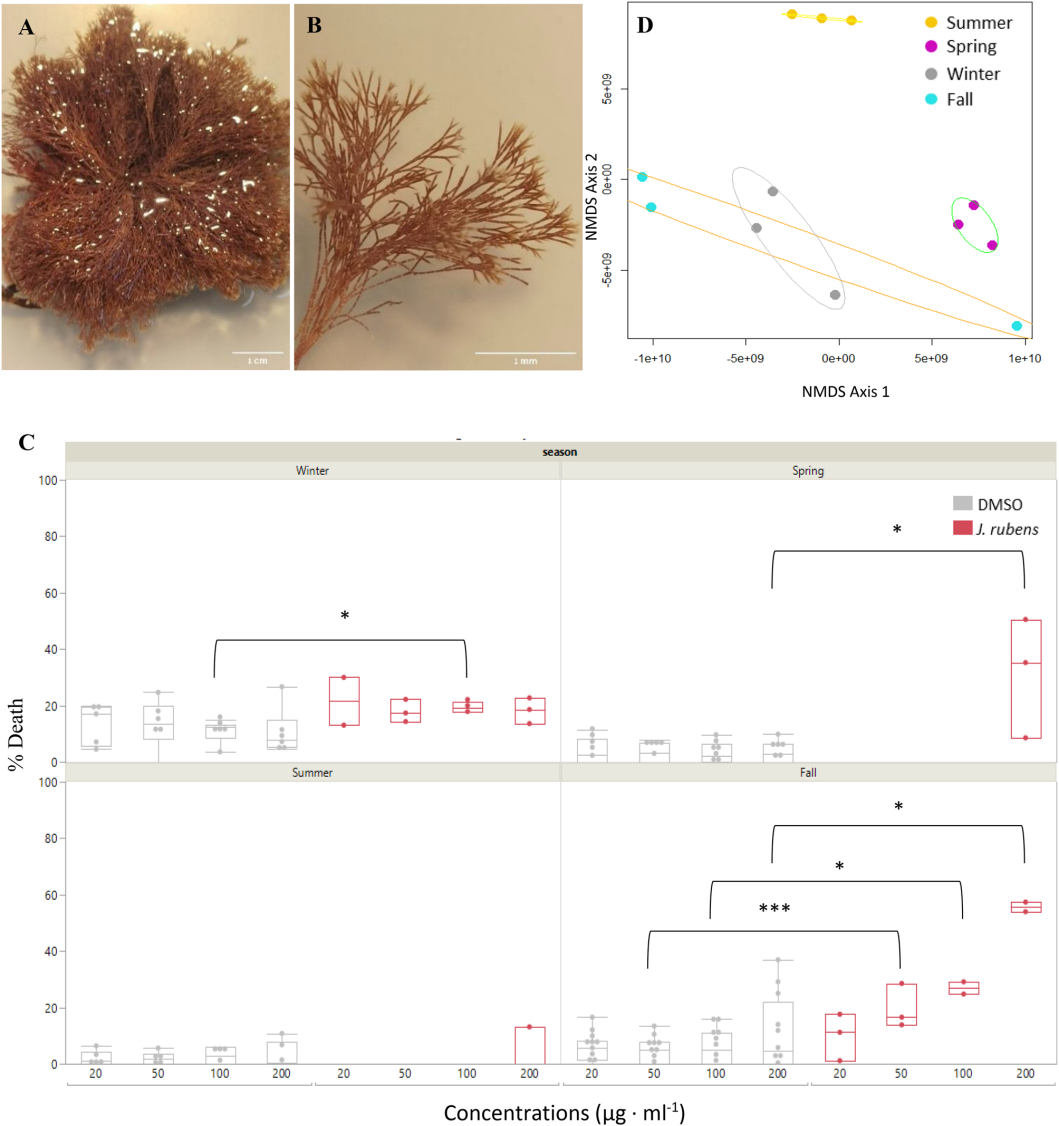
Seasonal variation in metabolic profile and anticancer bioactivity of *J*. *rubens*. Aerial (**A**) and closeup (**B**) view of *J. rubens* showing the characteristic dense clusters of branchy segmented cylindered thallus. (**C**) The cytotoxic activity of *J. rubens* methanolic extracts from each season toward A549 cells after 48 h incubation was assessed via microscopy imaging of Hoechst and PI staining and statistically analyzed by Wilcoxon test. Each dot represents an average of 3 independent experiments and presented as mean ± SE (*P < 0.05, ***P < 0.001). (**D**) *J. rubens* seasonal metabolic variation displayed by an NMDS plot, each ellipse represents a hierarchical cluster analysis.

To test the seasonal variation in the metabolic profile and bioactivity of seaweeds from the intertidal zone, we performed an annual screening in which we concurrently tested both. As part of this work, we identified *J. rubens* as a potential candidate for seaweed-based anti-cancer compounds. A significant change was observed in the bioactivity of *J. rubens* extracts in different seasons (Fig. 1A,B). Summer and Spring extracts showed poor cytotoxicity toward A549 cells and were only effective in the highest dose tested of 200 µg/ml, while Winter and Fall extracts demonstrated higher cytotoxic activity already at the starting dose of 20 µg/ml (Fig. 1C). The most potent activity was found for the extract of *J. rubens* samples collected in the Fall season, with an LC50 of 178.39 (± 10.02 SD) µg/ml, followed by the extract of samples collected in the Winter, which demonstrated a trend of cytotoxicity in all tested concentrations. To investigate these seasonal changes, we analyzed each extract by means of LC-MS/MS. Our analyses suggested the metabolic profile of the alga changed throughout the seasons (Fig. 1D).

### Biological effect of *J*.* rubens* extracts and fractions

**Figure 2:**
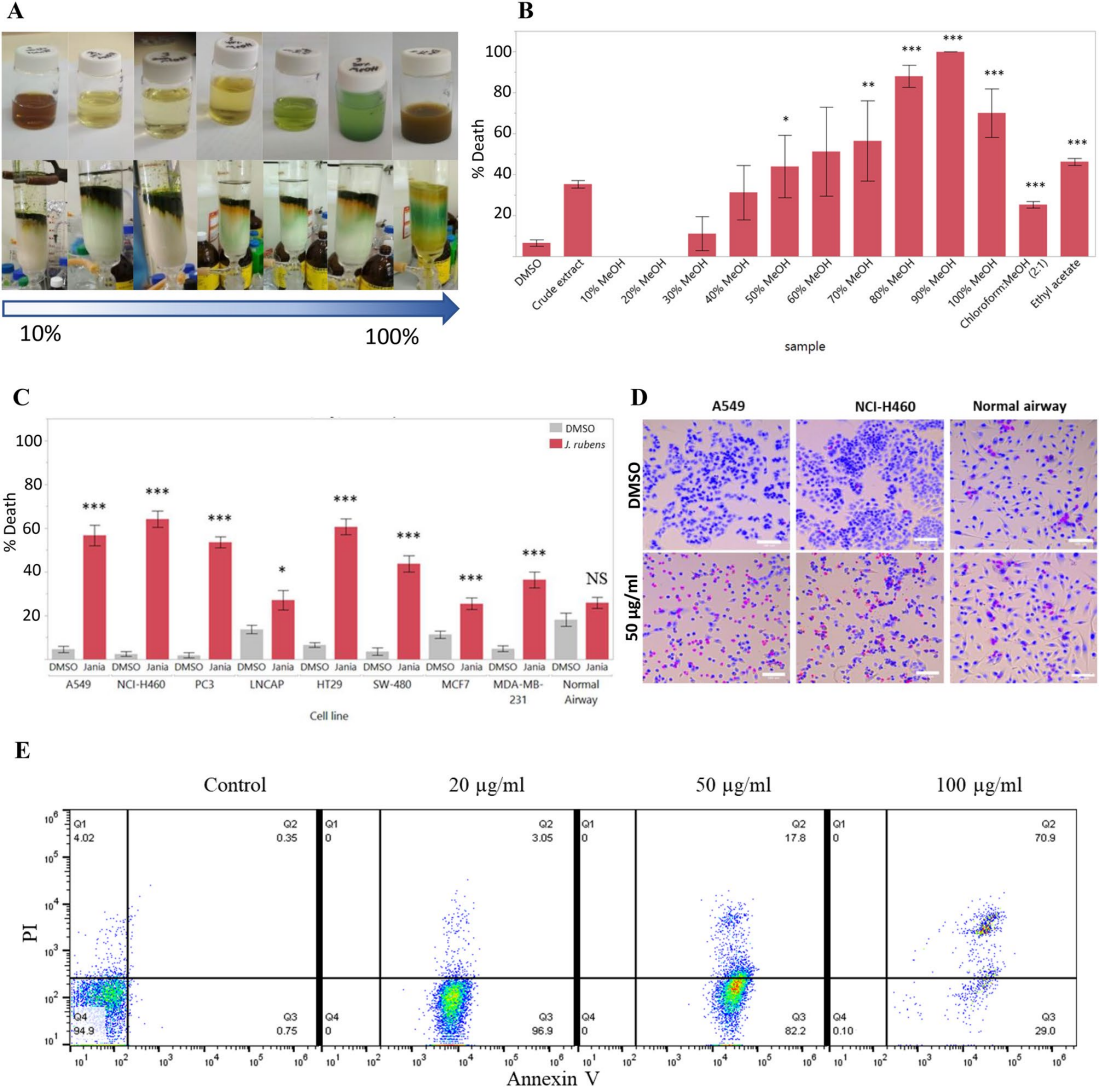
J. *rubens* fractions cytotoxicity measured in a cancer cell line array. (**A**) The *J. rubens* chloroform:MeOH (2:1 v/v) Fall extract underwent VLC chromatographic separation over silica RP and eluted with increased MeOH percentage, resulting in fractions of different colors. (**B**) A549 cells were treated with *J. rubens* Fall extract and every elution fraction at 100 µg/ml for 48 h (n = 2). Cell death is presented as mean ± SE. Statistical significance was analyzed with Wilcoxon test (*P < 0.05, **P < 0.01, ***P < 0.001). (**C**) Elutions 60 to 80% MeOH were pulled together and the resulting VLC80 was screened for its cytotoxic effect on A549, NCI-H460, PC3, lnCAP, HT29, SW480, MCF7, MDA-MB-231 and normal airway cells at 50 µg/ml after 48 h incubation. Data are presented as a mean of 3 biological repeats ± SE, and statistically analyzed relative to DMSO control with one-way ANOVA and followed by a Tukey HSD means comparison test (*P < 0.05, ***P < 0.001, NS – non-significant). (**D**) Representative microscopy image of A549, NCI-H460 and normal airway cell lines treated with DMSO or 50 µg/ml VLC80 and stained with Hoechst (blue) and PI (violet). Scale bars represent 100 µm. (**E**) A549 cells were incubated for 24 h with VLC80 at 20, 50, and 100 µg/ml and apoptosis was assessed relative to DMSO control by staining with Annexin-V/PI and analyzing the apoptotic rate via flow cytometry. The lower left quadrant contains the double negative population of vital cells, the lower right quadrant contains the early apoptotic stage (Annexin V + /PI −) population, the upper right quadrant contains the dead (annexin V + /PI +) cells population, and the upper left quadrant contains non-apoptotic dead cells (annexin V − /PI +).

As the *J. rubens* collected in the Fall showed the highest potency on A549 cell line, it was chosen for further characterization. To identify the active ingredients, several fractionation steps were used. The first fractionation step was carried over silica RP using a VLC column, with consecutive elution steps using an increased MeOH-to-water ratio, followed by chloroform:MeOH and ethyl acetate elutions (Fig. 2A). Fractions eluted in the range of 60%-80% MeOH induced A549 cell death at all tested concentrations (Fig. 2B). These elutions were pulled together and referred to from here on as VLC80. To test whether VLC80 can exert a cytotoxic effect on other cancer cell types, we conducted a screening of VLC80 in seven concentrations ranging between 2 and 200 µg/ml (Supplementary Fig. 1) on several cell lines: an additional NSCLC cell line, NCI-H460; two types of prostates adenocarcinoma cell lines, PC3 and lnCAP; two types of colorectal adenocarcinoma cell lines, HT29 and SW480; and two types of breast adenocarcinoma cell lines, MCF7 and MDA-MB-231. To assess the selectivity of VLC80 to cancer cells, we also tested its effect on a normal bronchial/tracheal epithelial cell line. At 100 µg/ml (Fig. 2C,D), NSCLC cells were the most susceptible to the treatment, with 56.73% ± 4.7SE and 64.13% ± 3.7SE cellular death for A549 and NCI-H460, respectively. The cells death recorded in the other cell lines in decreasing sensitivity to treatment was as follows: HT29 with 60.57% ± 3.5SE, PC3 (53.51% ± 2.5SE), SW480 (43.7% ± 3.7SE), MDA-MB-231 (36.43% ± 3.6SE), lnCaP (27.12% ± 434SE), and MCF7 with 25.45% ± 2.69SE cellular death. While all the cancer cell lines responded to the treatment with different degrees of severity, the normal epithelial cells were only affected by doses over 100 µg/ml of VLC80. This variation in cellular response suggested the specificity of VLC80 toward cancer cells, namely NSCLC, and also suggested the activation of a cellular death mechanism over an unspecific death response. As apoptosis is the common cellular death mechanism avoided by cancer cells thriving uncontrollably^31^, we tested whether VLC80 induced the apoptosis of A549 cells (Fig. 2E). Upon administration of 20 µg/ml VLC80, cells entered an early apoptosis stage, exhibiting high Annexin-V and low PI staining. With increasing doses, the cells entered a late apoptotic stage (positive for both Annexin-V and PI) and administration of 100 µg/ml VLC80 resulted in a dramatic drop in the number of viable cells.

### Isolation and identification of an active compound

**Figure 3:**
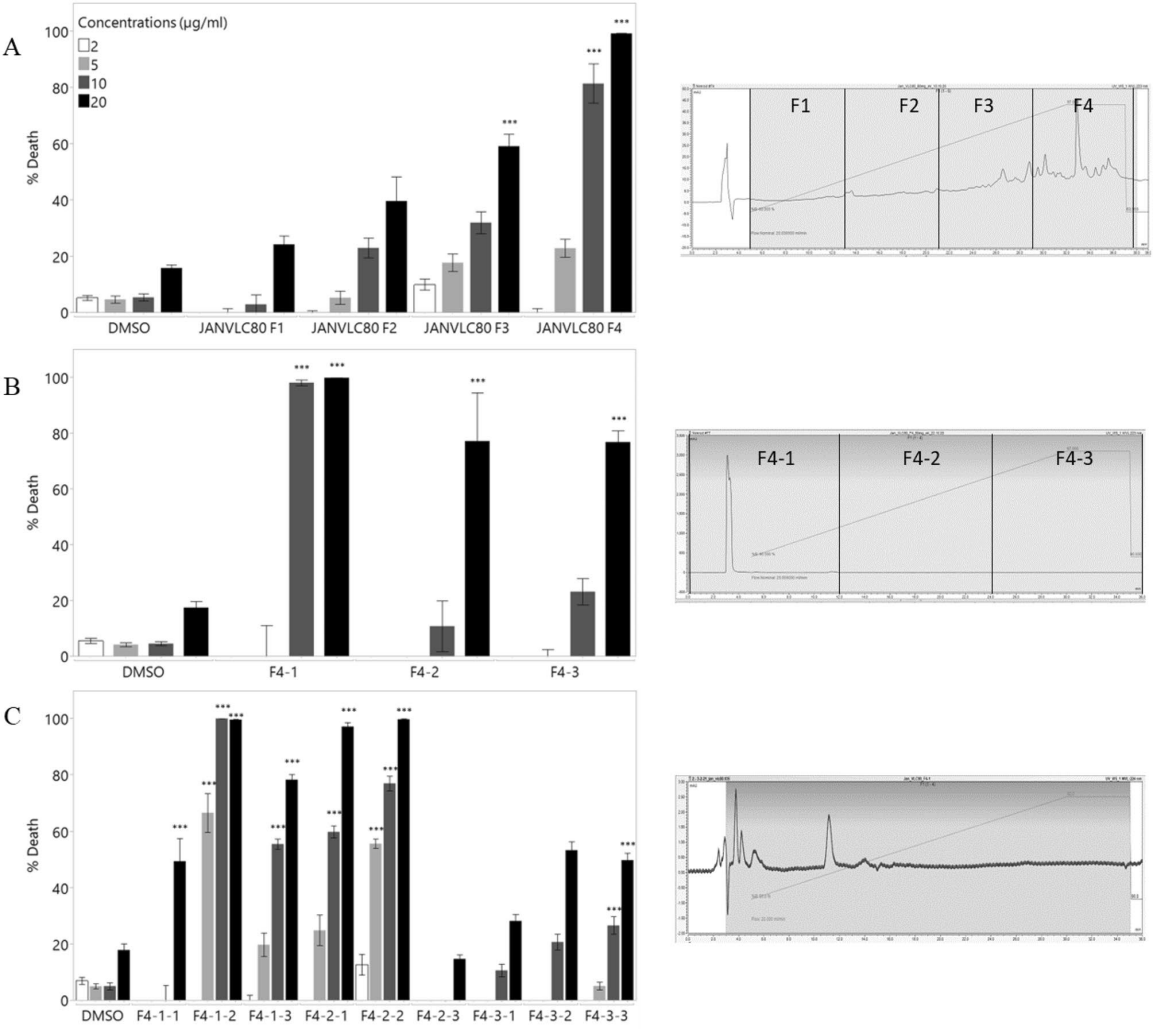
Consecutive fractions of VLC80. Fractions were tested for cytotoxic bioactivity on A549 cells after 48 h treatment and the most effective fraction underwent additional fractionation. (**A**) VLC80 was separated into four fractions F1-F4 and F4 showed the highest potency. (**B**) F4 was separated into three fractions. (**C**) The three F4-1 fractions were separated into three subfractions. Data were obtained from 3 biological repeats and presented as mean ± SE. Statistically analyzed with one-way ANOVA followed by a Tukey HSD means comparison test (***P < 0.001).

**Figure 4:**
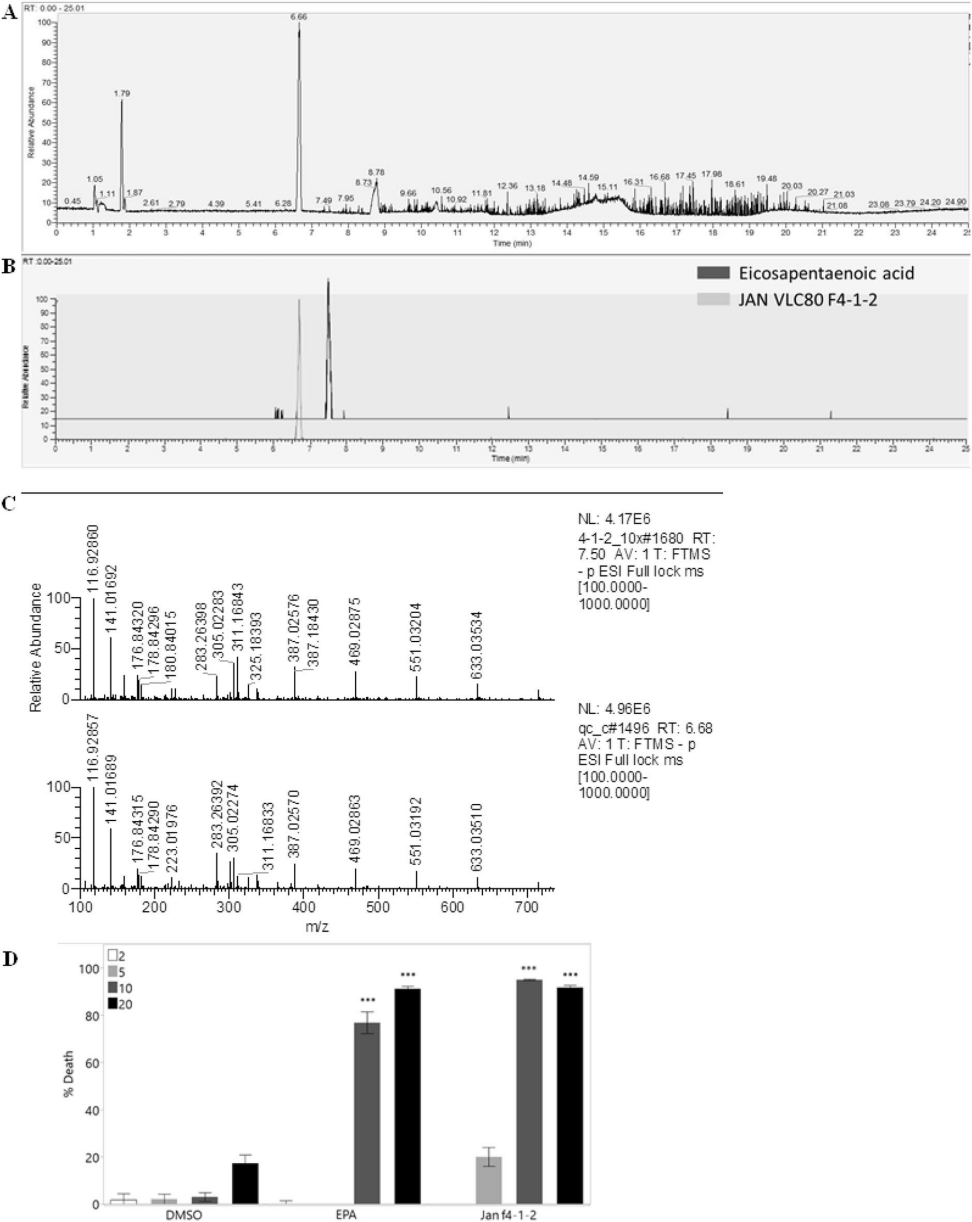
Spectral data and bioactivity of fraction F4-1-2. (**A**) Base peak results of LC-MS/MS for fraction F4-1-2. (**B**) Mass range for 302.2185 m/z results of LC-MS/MS for fraction F4-1-2 and an EPA standard. (**C**) Mass spectral comparison between fraction F4-1-2 and EPA standard according to LC-MS/MS, observed for mass range of 302.2184 m/z. (**D**) Comparison of biological effect between fraction F4-1-2 and EPA standard after 48 h incubation on A549 cell line, compared to DMSO control. Data were obtained from 3 biological repeats ± SE and statistically analyzed with two-way ANOVA followed by a Tukey HSD means comparison test (***P < 0.001).

To isolate the active compounds in VLC80, we performed consecutive fractionations using semi-preparative HPLC (Fig. 3). Each fraction was tested for its cytotoxic effect on A549 cell line in four different concentrations and the most active fraction was further fractionated. Thus, fraction 4 (Fig. 3A) was further fractionated into three subfractions. Of them, fraction 4–1 (Fig. 3B) was further fractionated into three subfractions. Finally, of these three fractions, fractions F4-1-2 and F4-2-2 showed the highest cytotoxic effect on A549 cells (Fig. 3C). We analyzed the compounds in both fractions by means of LC-MS/MS (Fig. 4A). These fractions mainly consisted of a single large peak. We matched this peak to a NIST library and identified it as cis-5, 8, 11, 14, 17-eicosapentaenoic acid methyl ester (EPA). To verify our results, we compared fraction F4-1–2 to a commercial standard of EPA and revealed the active metabolite from *J. rubens* is an isomer of EPA, as shown by the shift in retention time (Fig. 4B,C). In order to validate that EPA is the molecule responsible for the cytotoxicity of J. *rubens* extract on A549 cells, we used synthetic commercial EPA and compared its activity to *J. rubens* fraction F4-1-2 of. First, we quantified the EPA in F4-1-2 by using a calibration curve of a commercial standard, it revealed that the fraction consisted of 71% EPA. Next, we compared the biological activity of fraction F4-1-2 to that of the synthetic commercial EPA (Fig. 4D). Both F4-1-2 and commercial EPA yielded similar percentages of cell death, showing an LC50 of 9.34 (± 0.19 SD) µg/ml and 5.23 (± 0.07 SD) µg/ml for the commercial and isolated EPA, respectively.

## Discussion

Plants of the marine environment, especially in the intertidal zone, are subjected to major shifts in both biotic and abiotic stresses such as changes in salinity and light radiation, herbivory and epiphytic settlement and contamination from marine and terrestrial sources. As such, their metabolic response must adjust to react to such changes. The nature of the stress cause is strongly controlled by seasonality. For example, some herbivores will be more abundant during certain seasons and some epiphytes might proliferate in the same manner. As *J. rubens* grows year-round, it faces many forms of grazing by fish, mollusks and crustacea, as well as the competition that rises due to seasonal dynamics of epiphyte algae and microbial settlements^32,33^. Considering that, our findings strengthen the assumption that seasonal variation can have a major impact on the chemical profile and bioactivity of the collected seaweed and therefore should be considered when one attempts to explore natural compounds with biological effects.

The marine seaweed *J. rubens* has been previously investigated for its anti-cancer properties, it is an organism from which originated novel sterols and brominated diterpenes with anticancer activity^19,20^. Yet, in this research we have identified an essential PUFA, EPA, to be the active compound in our isolates. As terrestrial higher plants do not have the capability to synthesize long-chain PUFAs such as EPA^34^, EPA nutrition can be obtained either by consumption of α-linolenic, a precursor for long-chain fatty acids synthesis in mammalians which can be found in terrestrial plants, or by its direct consumption from marine sources such as micro and macro algae and fish^35,36^. EPA has previously demonstrated a seasonal pattern in many marine organisms^37–39^ with a strong correlation between cold temperatures and an increase in PUFA content^39–41^. This trend of seasonal variation in the concentration of EPA was also reported in marine seaweeds^42,43^, including *J. rubens*. The EPA content of *J. rubens* was estimated to range between 9 and 24.47% of total fatty acids^15,44^, which translates to about 7% of the seaweed dry weight^45^, and to change seasonally with the reported peak concentration depending upon the research location. As our Fall sampling in Israel during November–December was conducted during a temperature drop (Supplementary Fig. 2), it can explain the increase in PUFA content, namely EPA, which correlates with the increased cytotoxicity we observed during the Fall and Winter seasons.

EPA and other omega-3 PUFAs are important for human health and are crucial in many biological processes. They are the precursors of eicosanoids such as prostaglandins, thromboxanes, and leukotrienes^46^ that possess protective qualities against the development of cardiovascular diseases, inflammation, atopic dermatitis, psoriasis, and other malignancies^47,48^. Studies in the past decade suggested omega-3 PUFAs such as EPA also play a crucial role in cancer metabolism.

The anticancer effects of EPA on NSCLC, and A549 cells in particular, were previously reported and are well established. These studies suggest the mechanism via which EPA suppresses proliferation and induces apoptosis of A549 cells is by enhancing the formation of autophagosomes^49^, organelles involved in recycling damaged components^50^. Other studies suggested the mechanism of action of EPA in NSCLC cells involves a shift in prostaglandins, enhancing the formation of the anti-inflammatory PGE3 at the expense of pro-inflammatory PGE2, which leads to a reduction in inflammation, metastasis, proliferation and angiogenesis in cancer cell lines^51,52^. Our results are in line with previous research that explored the apoptotic impact of EPA on the A549 cell line and its specific effectiveness against cancer cell lines^49,50,53,54^. In this study, the EPA from *J. rubens* was isolated and identified through LC-MS/MS analysis, compared with a NIST mass spectral library, and validated using an EPA standard, which showed similar LC50 values. The VLC80 fraction has shown increased potency against NCSLC cell lines and induced apoptosis in A549 cell line after 24 h treatment. The slightly enhanced bioactivity of the isolated EPA relative to the commercial one might be attributed to the isolated EPA being an isomer, necessitating further exploration of the distinctions between invertebrate processed and natural seaweed-originated EPA forms, and might be of interest for understanding the importance and health implications of PUFA consumed in modern diet^55^.

EPA is important in human nutrition. In the western diet, essential PUFAs are obtained by consuming fish products which, beforehand, consumed and accumulated essential PUFAs from plant sources such as micro- and macro-algae. Although *J. rubens* is widely abundant in nature, to utilize its high content of PUFA and EPA^45^ for industrial purposes, efforts should be directed to its cultivation to avoid damage of this important marine costal habitat. PUFAs have an important role in cell membrane fluidity and are important for cell functioning in cold environments^56^. Therefore, PUFAs are known to increase in plants during cold seasons. Similarly, previous reports showed the EPA constitute in marine algae increased during cold seasons^57^. These findings correlate with our results showing increased cytotoxicity of *J. rubens* during the Fall and Winter seasons. Along with its high abundance and robust growth, our findings put forward *J. rubens* as a potential source organism for omega-3 PUFAs and EPA and might raise an alternative to the current commercial fish based PUFAs.

### Supplementary Information

Supplementary Figure S1.

Supplementary Figure S2.

## Data Availability

The authors confirm that the data supporting the findings of this study are available within the article and its supplementary materials. Datasets accompanying this research can be found in figshare repository, 10.6084/m9.figshare.22793627.

## Abbreviations

EPA: Eicosapentaenoic acid
*J. ruben*: *Jania* *rubens*
NSCLC: Non-small cell lung cancer
PUFA: Poly unsaturated fatty acid
MeOH: Methanol
LC-MS: Liquid chromatography-mass spectrometry
UHPLC: Ultra-high-performance liquid chromatography
HRMS: High resolution mass spectrometry
VLC: Vertical liquid chromatography
NIST: National Institute of Standards and Technology
