# Supplementary material for: Seasonal variation in the metabolome expression of Jania rubens (Rhodophyta) reveals eicosapentaenoic acid as a potential anticancer metabolite
Source: Sci Rep. 2023 Sep 20;13:15559. doi: 10.1038/s41598-023-42497-0 (PMC10511708; doi:10.1038/s41598-023-42497-0)
Supplement: Supplementary file 1 — Supplementary Figure S1. [file 41598_2023_42497_MOESM1_ESM.pdf]

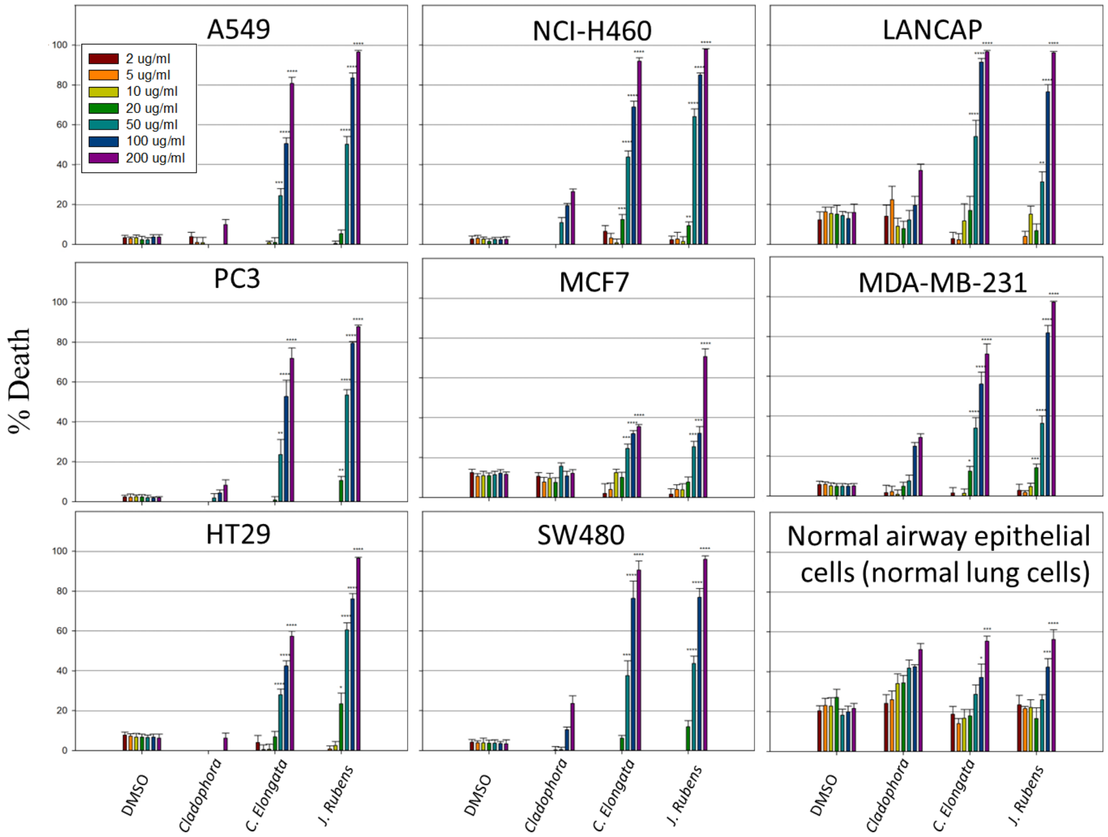

Figure S1- Screening results of VLC80 toward A549, NCI-H460, PC3, lnCAP, HT29, SW480, MCF7, and MDA-MB-231 at 2-200 µg · ml^{-1} after 48 hrs incubation. Data obtained from 3 biological repeats and presented as mean ± SE. Statistically analyzed with one-way ANOVA followed by a Tukey HSD means comparison test (*P < 0.05, **P < 0.01, ***P < 0.001, ****P < 0.0001).
