# Supplementary figures and images for: Seasonal variation in the metabolome expression of Jania rubens (Rhodophyta) reveals eicosapentaenoic acid as a potential anticancer metabolite
Source: Sci Rep. 2023 Sep 20;13:15559. doi: 10.1038/s41598-023-42497-0 (PMC10511708; doi:10.1038/s41598-023-42497-0)

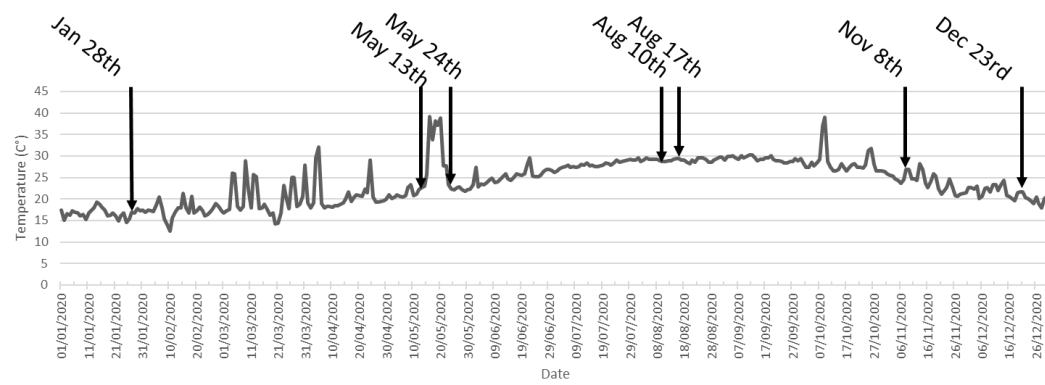

Figure S2- Collection dates and atmospheric temperature throughout the sampling period.

Supplement: Supplementary file 2 — Supplementary Figure S2. [file 41598_2023_42497_MOESM2_ESM.pdf]
